# Determinants of Prosocial Behavior in Included Versus Excluded Contexts

**DOI:** 10.3389/fpsyg.2015.02001

**Published:** 2016-01-07

**Authors:** Esther Cuadrado, Carmen Tabernero, Wolfgang Steinel

**Affiliations:** ^1^Department of Psychology, Social Psychology, University of CórdobaCórdoba, Spain; ^2^Department of Social and Organizational Psychology, Leiden UniversityLeiden, Netherlands

**Keywords:** prosocial behavior, exclusion, psychosocial variables, predictive model, mediation

## Abstract

Prosocial behavior (PSB) is increasingly becoming necessary as more and more individuals experience exclusion. In this context it is important to understand the motivational determinants of PSB. Here we report two experiments which analyzed the influence of dispositional (prosocialness; rejection sensitivity) and motivational variables (prosocial self-efficacy; prosocial collective efficacy; trust; anger; social affiliation motivation) on PSB under neutral contexts (Study 1), and once under inclusion or exclusion conditions (Study 2). Both studies provided evidence for the predicted mediation of PSB. Results in both neutral and inclusion and exclusion conditions supported our predictive model of PSB. In the model dispositional variables predicted motivational variables, which in turn predicted PSB. We showed that the investigated variables predicted PSB; this suggests that to promote PSB one could (1) foster prosocialness, prosocial self and collective efficacy, trust in others and affiliation motivation and (2) try to reduce negative feelings and the tendency to dread rejection in an attempt to reduce the negative impact that these variables have on PSB. Moreover, the few differences that emerged in the model between the inclusion and exclusion contexts suggested that in interventions with excluded individuals special care emphasis should be placed on addressing rejection sensitivity and lack of trust.

## Introduction

Civic cooperation, assistance, and solidarity are increasingly becoming necessary. More and more individuals are experiencing social exclusion resulting, for example, in job loss, eviction from one’s home or complete marginalization. Promotion of prosocial behavior (PSB) — defined as an broad range of acts, including helping behavior, altruism, cooperation and solidarity intended to benefit other people (Weinstein and Ryan, 2010) — in individuals, groups and communities encourages the development of networks that facilitate coexistence, well-being and healthier social and environmental contexts. It therefore seems important to analyze the motivational determinants of PSB. In this research we analyzed the influence of psychosocial variables — some dispositional and some motivational — on PSB, first in a neutral context (Study 1), and then in the context of included versus excluded groups (Study 2).

Based on the Cognitive Affective Personality System Theory (CAPS; Mischel and Shoda, 1995; Mendoza-Denton et al., 2001), we analyzed the role of some dispositional and psychosocial variables in predicting PSB in both a neutral and an inclusion versus inclusion contexts, as well as the potential relations between those predictors themselves. The CAPS conceives of the individual as a complex processing system, and suggests that the situation and the cognitive, affective, and personality components interact together, leading individuals to behave in a specific way. Thus, as state in the CAPS (Cervone, 2005), we proposed that some knowledge structures (the dispositional variables proposed in both studies) causally influence appraisal processes (as the psychosocial variables explored in both studies); that both kinds of variables interact together; and that this interaction leads individuals to behave in a specific way, i.e., in a prosocial specific way, as we are interested in explored in this study.

Shoda and Mischel (2006) claimed that the selection of the plausible mediators and determinants of a specific behavior depends on the behavior one is interested in predicting and on the situation within which this behavior is expected to occur. Thus, some variables widely studied in the past in relation to PSB (such as the dispositional and psychosocial variable of this study) seem to be potentially interconnected mediators that can be explored to predict this behavior by following the CAPS approach. Previous studies have shown that dispositional prosocialness (Eisenberg et al., 2002; Carlo et al., 2003), self-efficacy (Bandura, 2001; Caprara and Steca, 2005), and trust (Rotenberg et al., 2005; Welch et al., 2005; Derfler-Rozin et al., 2010; Berigan and Irwin, 2011) are potential predictors of PSB. Moreover, the relations between most of those variables have also been demonstrated, and therefore led us to theorize some meditational hypotheses not yet explored to our knowledge. Thus, it will be interested to explore the validity of a determinant model of PSB involving all those variables, by exploring how they interact together to explain PSB, what to our knowledge has not been explored to the date. Moreover, the exploration of some variables—as rejection sensitivity, anger and affiliation motivation—seems to be particularly relevant in order to explain PSB in the context of social inclusion. Thus, it may be relevant to explore the validity of the model explored in a context of social exclusion by adding those variables explicitly relevant in this context, and once more by exploring the relations those variables maintain between themselves and their potential mediating role in explaining PSB in such contexts.

In brief, the global aim of our two studies was to analyze the role of some dispositional and psychosocial variables in predicting PSB, and to analyze the relation between those predictors themselves by testing the potential mediating effects of self and collective efficacy, trust, anger and affiliation motivation, in accordance with the CAPS (Mischel and Shoda, 1995; Mendoza-Denton et al., 2001). The variables studied were chosen in line with the CAPS (Mischel and Shoda, 1995; Mendoza-Denton et al., 2001) that discuss interconnected mediators, which predict individual behavior. In line with the premise of Shoda and Mischel (2006), the relevance of one or other mediator depends in part on the behavior theorists are interested in predicting and on the context in which this behavior occurs. As such, the dispositional and psychosocial variables chosen for this study have been commonly related to PSB in previous literature and/or to social exclusion situations.

The potential of this study lies in the fact that it explores a potential model of PSB, including the potential relations between different dispositional and psychosocial variables, exploring not only the effect of those variables on PSB, but also the potential interactions between themselves; interactions that finally led to explain PSB. Moreover, we then apply this model to the context of social exclusion versus social inclusion by adding some variables especially relevant in those contexts. In this sense, the analysis of such variables as predictors of PSB, and the testing of the potential relations between them, may be pertinent from a theoretical perspective. Additionally, from an applied perspective, because social exclusion is a common result of the crisis, and because social assistance and PSB promote healthier social and environmental contexts and thus are increasingly necessary, it seems relevant to study which variables can be predictors of PSB, not only in neutral contexts, but also in the contexts of inclusion versus exclusion situations; and then propose some practical interventions based on the results to promote this kinds of beneficial behaviors.

### Psychosocial Variables Related to Prosocial Behavior

Many variables have been related to PSB. Dispositional prosocialness, i.e., the disposition or tendency to help, share, cooperate, empathize and take care of other people (Caprara et al., 2000) might be a predictor of PSB. It has been demonstrated that (1) prosocial tendencies correlate positively with global PSB and negatively with aggression (Carlo et al., 2003), (2) prosocial disposition in childhood is related to PSB in young adulthood (Eisenberg et al., 2002) and (3) that individuals with prosocial orientation engage in more PSB, e.g., donating than individuals with individualistic and competitive orientations (Van Lange et al., 2007). Additionally, it is assumed that individuals’ behavior tends to be congruent with their disposition (Heider, 1958) and that attitudes drive behavior (Helper and Albarracin, 2014) i.e., a positive attitude to some object or objective will result in behavior designed to increase or promote it. We therefore argue that prosocialness will predict PSB.

H1: Individuals with higher levels of prosocialness engage in higher levels of PSB.

### Prosocial Self-Efficacy and Prosocial Collective Efficacy and Related Variables

Self-efficacy can be responsible for unity and directness in terms of the individual’s actions (Caprara and Steca, 2005). The relationship between behavior and perceived efficacy — at both individual and collective level —has been widely debated (for a review see Bandura, 2001). Without confidence in their ability or the ability of their group to do something, it is unlikely that individuals will engage in a related behavior (Bandura, 2001). There is also evidence that empathic self-efficacy directly predicts PSB across ages (Caprara and Steca, 2005). From this evidence it follows that higher prosocial self-efficacy — confidence in one’s own ability to act prosocially — and higher collective prosocial efficacy — confidence in the ability of one’s group to act prosocially — will predict higher levels of PSB (Cuadrado and Tabernero, 2015).

Prosocialness has been associated with self-efficacy. Highly prosocial individuals probably tend to have high levels of confidence in their ability to behave in a prosocial way. Bandura et al. (1999) confirmed the relationship between prosocialness and both self-efficacy and social efficacy. The relationship between empathic self-efficacy beliefs and prosocialness is dynamic (Alessandri et al., 2009). Hence, it seems that the greater the prosocialness levels individuals possess, the more their prosocial self-efficacy will be elevated.

Prosocial self-efficacy and collective prosocial efficacy are also related. Self-efficacy influences beliefs about the effectiveness of one’s group (Fernández-Ballesteros et al., 2002). In other words individuals who doubt their own efficacy probably have little confidence in the efficacy of their group, and vice versa (Bandura, 2000; Fernández-Ballesteros et al., 2002).

H2: Prosocial self-efficacy mediates the relationship between (a) prosocialness and prosocial collective efficacy, and (b) prosocialness and PSB.

### Trust and Related Variables

Previous research has shown that trust, which “represents confidence in the strength of a partner’s commitment” (Rusbult and Agnew, 2010, p. 339), promotes PSB (Rotenberg et al., 2005; Welch et al., 2005; Derfler-Rozin et al., 2010; Berigan and Irwin, 2011).

It is easy to understand the relationship between prosocialness and trust: prosocial individuals expect that PSB will be reciprocated and therefore tend to trust others. The more empathetic an individual is — empathy is an important component of prosocialness (Caprara et al., 2005) — the more likely it is that he or she will feel something in common with others and therefore the more likely he or she is to trust others (Levenson and Ruef, 1992) and be willing to approach them. Empathy and prosocialness promote good interpersonal relationships (Davis and Oathout, 1992) and it has been claimed that empathy and trust are closely related (Ickes et al., 1990). Feng et al. (2004) showed that in an online context empathic communication increases trust. Altruism, benevolence, and generosity — which are strongly associated with prosocialness — have also been found to predict trust (Nooteboom and Six, 2003; Klapwijk and Van Lange, 2009; De Dreu et al., 2010). For example, Klapwijk and Van Lange (2009) found that generosity has an important role in building and maintaining trust; and De Dreu et al. (2010) found that parochial altruism promoted in-group trust. We anticipated that more prosocial individuals would show more trust.

It also seems likely that individuals who believe strongly in the prosocialness of their group are confident that group members will treat them with goodwill and benevolence. Sapouna (2010) defined collective efficacy — which is strongly related to, and intertwined with trust (McKenzie et al., 2002) — as “a mutual trust (among the members of a group) combined with their willingness to intervene to achieve common goals” (p. 1920). This suggests that collective efficacy may play a critical role in decisions about the trustworthiness of group members (Kramer et al., 1996). De Cremer (1999) showed that high perceived collective efficacy reduced fear and thus enhanced individuals’ trust in the cooperative intentions of others. We anticipated that individuals with high collective prosocial efficacy would trust in the goodwill of their partners.

H3: Collective prosocial efficacy mediates the relationship between (a) prosocialness and trust, and (b) prosocial self-efficacy and trust.

H4: Trust mediates the relationship between prosocialness and PSB.

**FIGURE 1:**
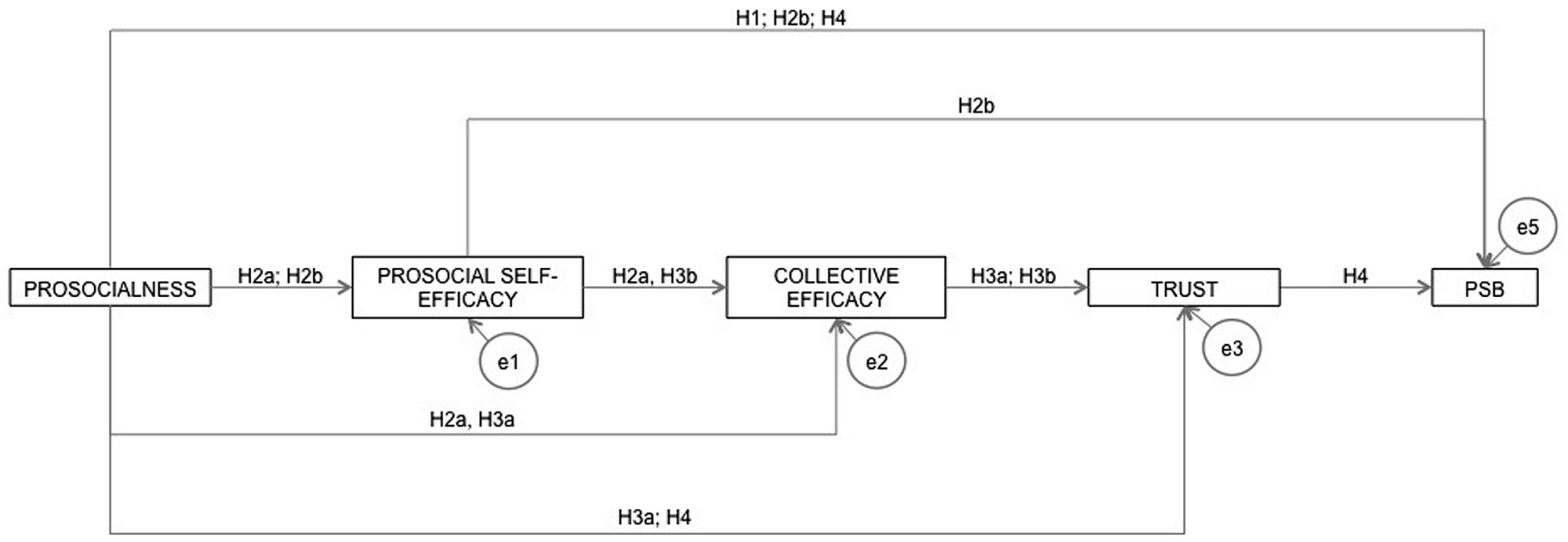
**Hypothesized predictive model of prosocial behavior; PSB, prosocial behavior**.

In short, as **Figure 1** shows, we proposed a predictive model of PSB in which prosocialness and trust were direct predictors of PSB; prosocial self-efficacy mediated the relationships between (1) prosocialness and prosocial collective efficacy and (2) prosocialness and PSB; collective efficacy mediated the relationships between (1) prosocialness and trust and (2) prosocial self-efficacy and trust; and trust mediated the relationship between prosocialness and PSB.

In Study 1 we tested this model in a neutral context. But what happens when individuals are suffering exclusion? Would the variables tested in this model still predict PSB? Would inclusion/exclusion moderate how predictive variables influenced PSB? Previous studies have shown that exclusion and inclusion can influence the extent to which an individual behaves prosocially (e.g., DeWall and Richman, 2011; Lee and Shrum, 2012), so in Study 2 we tested our model in two different conditions — inclusion and exclusion — adding some variables — rejection sensitivity, anger, and affiliation motivation—which seemed relevant to the context conditions.

## Study 1

The objective of this study was to analyze the relationships between the various motivational determinants of PSB and devise a predictive model of PSB in a neutral context.

### Materials and Methods

#### Participants, Measures and Procedure

The participants were 93 students (86% women, 14% men; age range: 21-43 years, *M* = 23.46, *SD* = 2.94) randomly selected from the University of Cordoba (Spain).

Students completed in our laboratory an online questionnaire created with the Global Park survey program. Then participants were informed that they would have to do some online group tasks in which they would have the opportunity to earn points, which would be exchanged for cash at the end of the experiment (this was part of the manipulation; there were no online participants). Before the group task, dispositional prosocialness was assessed. Then, to ensure the reliability of the online group tasks, the program asked participants to introduce themselves to the other online contestants. Then, in order to know the other participants who may comprise their group, they read the description of six participants (all the participants read the same descriptions of fictitious online participants. Descriptions gave information on sex, age, career choice, academic course, leisure interests etc.). At this point they were informed that the computer had randomly allocated them to a three-person online group. Next prosocial self-efficacy, collective prosocial efficacy, and trust were assessed. After this the participants played three rounds of the public good dilemma game; this allowed them to earn points that could be exchanged for cash (all participants were informed that they had earned 10 euros). Finally, participants were fully debriefed and probed for suspicion.

The Spanish Ministry of Economy and Competitiveness only requires revision and approval by an institutional review board (ethics committee) when the studies imply (a) clinical human experimentation; (b) use of human embryonic stem cells, or derived therefrom, from pre-embryos remaining lines; (c) Use of tissues or biological samples of human origin; (d) Use of personal data, genetic information, etc.; (e) Animal Experimentation; (f) Use of biological agents of risk to human health, animal or plant; (g) Use of genetically modified organisms (GMOs); or (h) Release of GMOs. Thus, the study was not reviewed nor approved by any institutional ethics committee before the study began because it was exempt from ethical approval procedures.

##### Dispositional prosocialness

Prosocialness was measured with the short version of the Prosocialness scale (Caprara et al., 2005). This consists of 12 items, e.g., ‘I try to console people who are sad’ with responses given on a seven-point Likert scale.

*Prosocial self-efficacy [α = 0.88, *M* = 6.10, *SD* = 0.80, range (4.00–7.00)]*. Self-efficacy with respect to PSB was assessed using a short (five-items; ‘I can behave cooperatively,’ ‘I can distribute resources equitably,’ ‘I can make an equal division of a common monetary fund,’ ‘I can adopt behavior oriented to help others,’ and ‘I can share resources’) scale with responses given on a 7-point Likert scale, in accordance with Bandura’s (2006) guide to constructing self-efficacy scales . Because this was not a validated scale, we performed Exploratory Factorial Analysis (EFA) with Varimax rotation; this confirmed that the scale had a one-factor structure that explained 68.31% of the variance in scores.

*Prosocial collective efficacy [α = 0.94, *M* = 5.88, *SD* = 1.05, range (1.00-7.00)]*. Participants’ perceptions of the prosocial efficacy of their group were assessed with a short scale designed in accordance with Bandura’s (2006) guide to constructing self-efficacy scales. The scale consisted of the same five items as the individual prosocial self-efficacy scale and responses were given on the same 7-point Likert scale, but all the items were preceded by the phrase ‘My group can’ (e.g., ‘My group can behave cooperatively’). EFA with Varimax rotation confirmed that the scale had a one-factor structure that explained 83.16% of the variance in scores.

*Trust [α = 0.72, *M* = 5.22, *SD* = 1.44, range (1.00-7.00)]*. Trust was assessed using an adaptation of Greenhalgh and Chapman’s (1998) scale. The scale included three items (e.g., ‘I feel that those two people can be counted on to help me’) to which participants responded using a 7-point Likert scale to indicate their trust in the participants with whom they were to perform the online group tasks. Participants completed the scale before solving the online group tasks. EFA with Varimax rotation confirmed that the scale had a one-factor structure that explained 77.43% of the variance in scores.

*Prosocial behavior [α = 0.88, *M* = 5.14, *SD* = 1.74, range (0.00-6.67)]*. PSB was assessed using the public good dilemma game; this in an *N*-person prisoner’s dilemma game which is usually used to assess tendency to cooperation. An explanation of the game by Santos et al. (2008, p. 213) states that “cooperators (C) contribute an amount c (‘cost’) to the public good; defectors (D) do not contribute. The total contribution is multiplied by an enhancement factor *r* and the result is equally distributed between all *N* members of the group.” In our experiment we used a three-person prisoner’s dilemma and three rounds were played. In each round players were given a certain number of points and had to decide how many points to keep and how many to donate. Donated points were doubled and distributed among the group. The mean number of points a participant donated over the three rounds of the game was used as a measure of PSB, donating more points indicated greater prosocialness.

#### Treatment of the Data

Sex and age were not the principal aim of our study and did not show any significant influence on the other variables of the study, and were thus omitted from all further analyses.

##### Preliminary analyses

In order to test the means and standard deviations of the variables of the study, as well as the interactions between them some descriptive analyses and correlation tests including all the variables were performed.

##### Multicollinearity tests

To detect multicollinearity we examined the correlation matrix for the independent variables, the variance inflation factor (VIF) and tolerance values for all the constructs (Kline, 2005).

##### Mediation analyses

In order to confirm hypotheses 2, 3, and 4 mediation analyses were computed with Amos (version 21) by following the product-of-coefficients strategy with bootstrapping to test the strength and significance of the indirect effect (Shrout and Bolger, 2002). In the present study the 95% confidence interval of the indirect effect was obtained with 2,000 bootstrap resamples.

##### Structural Equation Modelling (SEM)

In order to confirm a predictive model of PSB a path analysis was performed with Amos 21. To estimate the causal model the following indicators of the goodness of fit were used:

(a) Root Mean Square Error of Approximation (RMSEA), which is considered as a good fit with values lower than 0.05, as an adequate fit with values between 0.05 and 0.08, as a mediocre fit with values between 0.08 and 0.10, and as a not acceptable fit with values higher than 0.10. (Browne and Cudeck, 1993; Schermelleh-Engel and Moosbrugger, 2003);
(b) Comparative Fit Index (CFI) which is suitable if you have values above 0.97 (Schermelleh-Engel and Moosbrugger, 2003);
(c) Goodness of Fit Index (GFI), for which Hoyle (1995) suggests values above 0.9 as appropriate, and Schermelleh-Engel and Moosbrugger (2003) suggest values above 0.95 indicative of good fit.

### Results

#### Preliminary Analyses

Correlation analysis was used to explore the relationships among all investigated variables in the study. As can be seen in **Table 1**, all correlations were in the expected direction.

**Table 1:** Correlations, means, standard deviations, and alpha reliabilities for all the study one variables.

	1	2	3	4	5	Mean	Range	*SD*	α
(1) Prosocialness	-					5.96	(3.42–7.00)	0.72	0.90
(2) PS self-efficacy	0.64^∗∗^	-				6.10	(4.00–7.00)	0.80	0.88
(3) Collective PS efficacy	0.48^∗∗∗^	0.59^∗∗∗^	-			5.88	(1.00–7.00)	1.05	0.94
(4) Trust	0.41^∗∗∗^	0.25^∗^	0.48^∗∗∗^	-		5.22	(1.00–7.00)	1.44	0.72
(5) PSB	0.29^∗∗^	0.33^∗∗∗^	0.31^∗∗^	0.30^∗∗^	-	5.14	(0.00–6.67)	1.74	0.88

*^∗^*p <* 0.05, *^∗∗^p <* 0.01, *^∗∗∗^p <* 0.001*.

*PS self-efficacy, prosocial self-efficacy; collective PS efficacy, collective prosocial efficacy; PSB, prosocial behavior*.

#### A Predictive Model of Prosocial Behavior

To detect multicollinearity we first examined the correlation matrix for the independent variables; the absence of high correlations (i.e., 0.85 or greater) suggested that the data were not affected by collinearity (Kline, 2005). As **Table 1** shows, the highest correlation was between prosocialness and prosocial self-efficacy (*r* = 0.64). We next checked the VIF and tolerance values for all the constructs. All VIF values were less than 5.0 (range: 1.309-1.871) and all tolerance values were between 0.10 and1.0 (range: 0.535-0.764) so we can be confident that the data were not affected by multicollinearity (Kline, 2005).

Mediation hypotheses (H2, H3, and H4) were tested using bootstrapping analyses in Amos 21. As **Table 2** shows, all the hypotheses were confirmed.

**Table 2:** Type of Mediation Observed.

Hypothesis	Direct Beta without Mediator	Direct Beta with Mediator	Indirect Beta
H2a: PSness → PS self-efficacy → collective PS efficacy	0.48^∗∗∗^	0.17 (ns)	0.31^∗∗∗^
H2b: PSness → PS self-efficacy → PSB	0.29^∗∗^	0.13 (ns)	0.16^∗∗^
H3a: PSness → collective PS efficacy → trust	0.41^∗∗∗^	0.23 (ns)	0.18^∗∗^
H3b: PS self-efficacy → collective PS efficacy → trust	0.33^∗∗∗^	-0.09 (ns)	0.30^∗∗∗^
H4: PSness → trust → PSB	0.29^∗∗^	0.20 (ns)	0.09^∗^

*Direct and indirect effects calculated with bootstrapping analysis*.

*PSness, prosocialness; PS self-efficacy, prosocial self-efficacy; collective PS efficacy, collective prosocial efficacy; PSB, prosocial behavior. The first column is a statement of the hypothesis. The second column gives the regression weight for the direct association between the independent variable (IV) and the dependent variable (DV) before controlling for the effects of the putative mediator (M). The third column gives an estimate of the standardized direct effect of the IV on the DV after controlling for the effects of the putative M. The fourth column gives an estimate of the standardized indirect effect of the IV on the DV after controlling for the effects of the putative M in bootstrapping analysis*.

*^∗^*p* < 0.05, ^∗∗^*p* < 0.01, ^∗∗∗^*p* < 0.001*.

**FIGURE 2:**
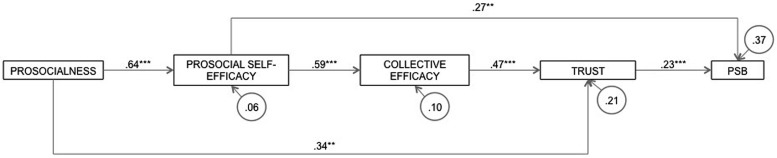
**Confirmed predictive model of prosocial behavior**. Values for relationships between variables are beta coefficients. PSB, prosocial behavior (^∗∗^*p* < 0.01, ^∗∗∗^*p* < 0.001).

Moreover, in order to confirm the predictive role of the variables, as well as the hypothesized predictive model of PSB, a path analysis was performed with Amos 21. The goodness-of-fit tests revealed that the model was well-fitted [χ^2^(3, *N* = 93) = 2.78, *p* = 0.43; *RMSEA* = 0.01 (95% *CI* [0.01,0.17]); *CFI* = 1.00; *GFI* = 0.99]. Results confirmed Hypotheses 2 (a and b), 3b—but not 3a—and 4, but only partially Hypothesis 1 (see **Figure 2**).

### Discussion

All the variables investigated contributed to a predictive model of PSB in which prosocial self-efficacy and trust act as direct predictors. The direct predictive role of prosocialness was not confirmed; it should, however, be noted that correlation and mediation analyses indicated that — in line with H1 — prosocialness was correlated with PSB and directly predicted it (*R* = 0.29^∗∗^; β = 0.29^∗∗^); although prosocialness was not a direct predictor of PSB in the model the two variables were related, with prosocialness directly predicting PSB. This result indicates that a prosocial disposition might lead individuals to behave prosocially, i.e., in congruence with their disposition (Eisenberg et al., 2002; Carlo et al., 2003).

Regarding the direct predictors of PSB and in line with previous studies (Rotenberg et al., 2005; Welch et al., 2005) the experiment showed that having confidence in partners’ goodwill encouraged individuals to behave in a prosocial way and conversely participants were less generous to partners they perceived as untrustworthy. Additionally, prosocial self-efficacy directly predicted PSB; the more confident individuals were in their ability to behave prosocially, the more likely they were to behave prosocially. This result is consistent with self-efficacy theory (Bandura, 2001), which states that individuals are less likely to attempt behaviors if they do not believe that they are capable of executing them successfully.

The mediating roles hypothesized were confirmed. Prosocial self-efficacy fully mediated the relationships between (1) prosocialness and prosocial collective efficacy and (2) prosocialness and PSB. The more prosocial an individual’s disposition the more likely he or she was to feel capable of behaving prosocially (Alessandri et al., 2009) and in turn, (1) the more they felt that their group was efficacious in behaving prosocially (Fernández-Ballesteros et al., 2002), and (2) the more they behaved prosocially (Bandura, 2001).

Prosocial collective efficacy fully mediated the relationships between (1) prosocialness and trust and (2) prosocial self-efficacy and trust. In accordance with previous research we found that (1) the greater individuals’ disposition to PSB the more likely they were to feel that their group was capable of behaving prosocially (Alessandri et al., 2009) and (2) the more individuals perceive themselves as highly efficacious in a determined behavior (being prosocial), the more they perceive that their group is efficacious in this same behavior (Bandura, 2000; Fernández-Ballesteros et al., 2002). In turn, the more they perceive their partner as having high abilities in being prosocial, the more they trust in those partners (Kramer et al., 1996; De Cremer, 1999).

Trust emerged as a mediator of the relationship between prosocialness and PSB. The more prosocial an individual’s disposition the more likely he or she is to trust others (Nooteboom and Six, 2003) and hence to behave prosocially toward them (Welch et al., 2005). This psychological pattern seems intuitively plausible: prosocial and empathic individuals usually see others like them, tend to expect some reciprocity, and consequently trust the others (Levenson and Ruef, 1992). In the expectation that the others will operate with goodwill, trust can produce not only reciprocity but also social orientation by bestowing on individuals the motivation to approximate those others, to engage in activities with them, as well as encouraging closeness as the starting-point for relationships (Welch et al., 2005); therefore it seems logical that trust may produce PSB (Baumeister and Leary, 1995; Zaskodna et al., 2013).

## Study 2

Study 1 provided evidence for a predictive model of PSB in which prosocialness, prosocial self-efficacy and trust act as predictors of PSB in a neutral context; however, previous studies have shown that exclusion and inclusion may affect the extent to which an individual behaves prosocially (Maner et al., 2007; Williams, 2007; Smart Richman and Leary, 2009; Romero-Canyas et al., 2010b; DeWall and Richman, 2011; Lee and Shrum, 2012). In our societies, more and more people are experiencing social exclusion, and even complete marginalization. In this context, the promotion of PSB is increasingly relevant. The causes of PSB have generally been attributed to positive experiences and factors; nevertheless PSB may also arise after negative life events (Vollhardt, 2009), as social exclusion. However, there is controversy about whether exclusion leads to prosocial (Maner et al., 2007; Mead et al., 2010) or antisocial behavior (Ayduk et al., 2008; Coyne et al., 2011). Consequently, it seems pertinent to explore how PSB is affected by inclusion and exclusion and whether the mechanisms that predict PSB in neutral contexts are the same in contexts of inclusion or exclusion. We were therefore interested in exploring potential contextual differences in associations between predictor variables and PSB; in particular we wanted to know whether the predictive variables explored in Study 1 were similarly powerful predictors of PSB in included and excluded individuals.

The objective of Study 2 was to determine if the model developed in Study 1 was valid for excluded and included individuals. We also added some supplementary variables of particular relevance to inclusion/exclusion contexts to the model: rejection sensitivity, anger, and affiliation motivation. In general, we expected that the variables that have shown to be predictors of PSB in Study One in a neutral context will be similarly powerful predictors of PSB in excluded and included contexts. There is no reason to think that prosocialness, prosocial self-efficacy, collective prosocial efficacy nor trust will not predict PSB in excluded and included contexts to the same extent as in a neutral context. Nevertheless, considering a new variables included in Study 2, it is interesting to note that we expected that rejection sensitivity will be predictor of anger and PSB only in contexts of exclusion, but not in contexts of inclusion. This prediction is based in the rejection sensitivity model of Levy et al. (2001) in which it is explained that rejection sensitivity is activated only when rejection cues are detected, triggering in turn negative affective states as anger, which in turn reduce the probability to behave prosocially.

### Psychosocial Variables Related to Prosocial Behavior

In Study 2 we used the model found in Study 1 is replicated by adding some variables of particular interest in the context of the exclusion-PSB relationship.

#### Rejection Sensitivity and Related Variables

Rejection sensitivity — i.e., the tendency to anxiously expect social rejection (Downey and Feldman, 1996) — moderates the link between exclusion and antisocial behavior: exclusion provokes aggression toward the rejecters in individuals who are highly sensitive to rejection but not in those who are less sensitive (Ayduk et al., 2008). Rejection sensitivity therefore seemed relevant to a model intended to predict PSB in the contexts of exclusion and inclusion.

Rejection sensitivity has been related to self-efficacy, which is in turn related to PSB. When rejection-sensitive individuals perceive rejection cues they activate negative self-efficacy beliefs (Ayduk et al., 2000). Rejection sensitivity impairs self-regulation, and — to an even greater extent — self-efficacy and interpersonal self-efficacy (Downey and Feldman, 1996; Levy et al., 2001; Inzlicht et al., 2006). The low interpersonal self-efficacy of high rejection sensitivity individuals produces decreases in confidence and skill in social interaction, particularly in the event of meeting new people, where there are more chances to be rejected; and as rejection sensitivity increases, interpersonal competence decreases (Butler et al., 2007). One would therefore expect rejection sensitivity to be negatively associated with prosocial self-efficacy and collective prosocial efficacy. We therefore predicted that:

H1: Prosocial self-efficacy mediates the relationship between rejection sensitivity and prosocial collective efficacy.

#### Anger and Related Variables

Anger increases when individuals feel excluded (Chow et al., 2008; Romero-Canyas et al., 2010b) and it has been shown that anger increases antisocial desires and exacerbates antisocial behavior (Leach et al., 2006) and reduces prosocial behavior in excluded individuals (Cuadrado et al., 2015). We consider that anger is relevant to models of the relationship between exclusion and PSB therefore included it as a motivational determinant in our predictive model of PSB.

There is evidence that anger is related to variables known to be associated with PSB, such as rejection sensitivity, collective efficacy and trust. In line with the rejection sensitivity model (Levy et al., 2001), Downey et al. (2000) offered a model in which—when rejection cues are perceived—high rejection sensitivity heightens cognitive-affective overreactions such as anger, that in turn increment the likelihood of violence occurring. In rejection-sensitive individuals exclusion elicits hostility (Ayduk et al., 1999) and reduces positive affect (Romero-Canyas et al., 2010b). Luterek et al. (2004) have also demonstrated that rejection sensitivity mediates the relationship between childhood sexual abuse and anger. We expected that the more individuals dread rejection, the more they feel angry when excluded.

Efficacy beliefs influence whether individuals think optimistically or pessimistically and their emotional responses (Bandura, 2000). Individuals who perceive that they or their group have low efficacy in a given task feel bad and activate a negative affect—such as anger (Valentino et al., 2009)—and a drop in positive affect (Salanova et al., 2011).

A propos trust, affective states influence the way in which we form an opinion of how trustworthy a person is (Jones and George, 1998). Individuals report more positive perceptions of others and report higher interpersonal trust when experiencing positive affect; conversely when experiencing negative affect, they are more likely to see others in a negative light and to perceive them as less trustworthy (Jones and George, 1998). Individuals experiencing positive affect tend to view human nature as more positive (Veitch and Griffitt, 1976), whilst anger decreases trust (Dunn and Schweitzer, 2005). We expected that angry individuals would trust their partners less.

In line with previous research and the results of Study 1 we hypothesized that:

H2: Anger mediates the relationship between (a) rejection sensitivity and trust, (b) prosocial self-efficacy and trust, and (c) prosocial collective efficacy and trust.

#### Affiliation Motivation and Related Variables

Affiliation motivation is the desire to maintain social contact or a sense of belonging (Veroff and Veroff, 1980); it motivates individuals to pursue positive interpersonal relationships (Zaskodna et al., 2013). High affiliation motivation reflects a strong sense of social interdependence (Markus and Kitayama, 1991) and so individuals with high affiliation motivation tend to act on behalf of their society or for the benefit of the group, i.e., in a prosocial manner. Individuals with high affiliation motivation will tend to behave in a friendly, prosocial manner in order to create or maintain social contact and avoid breaking bonds (Baumeister and Leary, 1995; Zaskodna et al., 2013). Many authors (Maner et al., 2007; Smart Richman and Leary, 2009; Romero-Canyas et al., 2010b; DeWall and Richman, 2011) have argued that rejected individuals tend to behave prosocially only when they see an opportunity to reconnect with others and have the desire to do so. These data suggested that affiliation motivation was likely to be a predictor of PSB.

There is also evidence that affiliation motivation is associated with several potential predictors of PSB. If we assume that prosocialness includes the tendency to take care of other people (Caprara et al., 2000) then it follows that prosocialness should increase desire for social contact and hence that prosocialness should predict affiliation motivation.

Rejection-sensitive individuals expect to be rejected by others and avoidance of such rejection is one of their primary goals (Downey and Feldman, 1996). Fear of rejection is an important component of affiliation motivation (Shipley and Veroff, 1958). Maner et al. (2010) argued that the increase in progesterone levels which is observed in individuals who dread rejection when they are given an opportunity to re-affiliate is consistent with their desire for compensatory social contact and their affiliation motivation. We anticipated that individuals who anxiously expect rejection would have a greater desire to continue interacting than less rejection-sensitive individuals.

The more capable individuals feel of doing something, the greater their motivation to act accordingly. Individuals who feel themselves to be highly capable of PSB are likely to behave prosocially, in accordance with this perception, and are more likely to be motivated to continue cooperating with partners than individuals with lower prosocial self-efficacy. This suggests that collective efficacy may increase the likelihood of engaging in relationships (Tasa et al., 2011). Social self-efficacy has also been related to the pursuit of social goals, as the more individuals feel socially efficacious, the more they endorse affiliation motivation (Patrick et al., 1997). We hypothesized that both self and collective prosocial efficacy would be positive predictors of affiliation motivation.

Given that trust is an expectation that others will contribute to positive outcomes and that trust tends to be reciprocal, individuals should have a greater desire to affiliate with people they trust. Trust leads to more open communication (Smith and Barclay, 1997) and to cooperation (Parks et al., 1996). Trusting individuals tend to be intrinsically motivated to engage in activities with others whereas less trusting individuals are less likely to want to affiliate (Green and Brock, 1998). Trust fosters closeness and is the starting point for personal relationships (Welch et al., 2005). We hypothesized that:

H3: Trust mediates the relationship between (a) prosocialness and affiliation motivation, and (b) prosocial collective efficacy and affiliation motivation.

H4: Affiliation motivation mediates the relationship between (a) prosocialness and PSB, (b) rejection sensitivity and PSB, (c) prosocial self-efficacy and PSB, (d) prosocial collective efficacy and PSB, and (e) trust and PSB.

**FIGURE 3:**
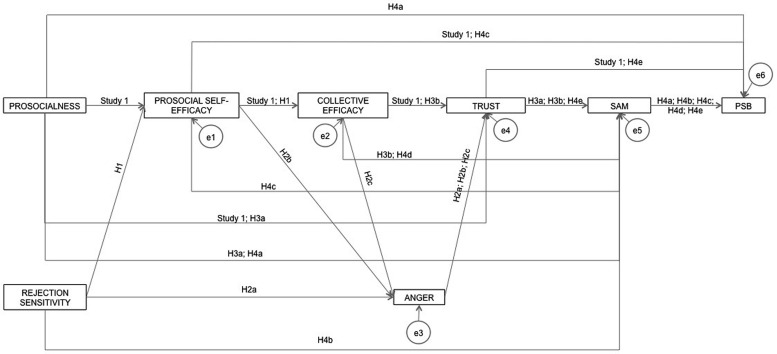
**Hypothesized predictive model of sharing resources prosocial behavior; SAM, social affiliation motivation; PSB, prosocial behavior - sharing resources**.

In short, our predictive model of PSB was very similar to that in Study 1, but included some supplementary variables. In this new model, in addition to the relationships of Study 1, prosocial self-efficacy also mediated the relationship between rejection sensitivity and prosocial collective efficacy. Anger mediated the relationships between (1) rejection sensitivity and trust, (2) prosocial self-efficacy and trust and (3) collective prosocial efficacy and trust. Trust mediated the relationships between (1) prosocialness and affiliation motivation and (2) collective efficacy and affiliation motivation. Affiliation motivation mediated the relationships between (1) prosocialness and PSB, (2) rejection sensitivity and PSB, (3) prosocial self-efficacy and PSB, (4) prosocial collective efficacy and PSB and (5) anger and PSB (see **Figure 3**).

### Materials and Methods

#### Participants

The participants were 119 students (71.4% women, 28.6% men; age range: 17-51 years, *M* = 19.89, *SD* = 5.18) randomly selected from the University of Cordoba (Spain). Students who take part in the first study were not able to take part in this second study.

#### Manipulation and Measures

The procedure was similar to that used in Study 1. Participants completed an online questionnaire in our lab and were then informed that they would have to do some online group tasks in which they would be able to earn points that would be exchanged for cash at the end of the experiment. Before the group tasks, dispositional prosocialness, rejection sensitivity and anger were assessed. Next, to ensure the reliability of the online group tasks, the program asked the participants to introduce themselves to the rest of the online contestants. Then participants read descriptions of six fictitious participants (all the participants read the same descriptions). They were then told that the computer had randomly allocated them to a three-person online group. At this point a sense of exclusion or inclusions was induced by having the participants play a round (30 passes in total) of the fourth version of the Cyberball game (Williams et al., 2012), a program developed for research on exclusion. Participants were randomly assigned to the exclusion condition (in which they received the ball only twice) or the inclusion condition (in which they received the ball ten times).

At this point a manipulation check was performed. Then, prosocial collective efficacy, anger, social affiliation motivation and trust were assessed. Then participants played two rounds of the *N*-person prisoner’s dilemma game [*M* = 2.58, *SD* = 0.78, range (0.00–3.50); *M*_included_ = 2.63, *SD* = 0.79, range (0.00–3.50); *M*_excluded_ = 2.52, *SD* = 0.77, range (0.00–3.50)] to assess PSB. After the two rounds, participants were informed that we had obtained enough data and that no further play was required. Finally, participants were fully debriefed and probed for suspicion.

As study one, this study was exempt from ethical approval procedures and thus was not reviewd nor approve by any institutional review board (ethics committee).

**FIGURE 4:**
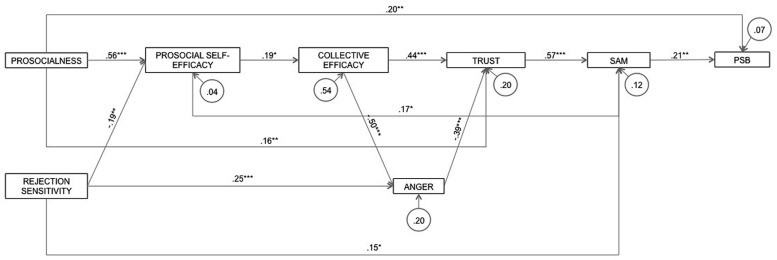
**Predictive model of sharing resources prosocial behavior confirmed to be equal across the two samples**. SAM, social affiliation motivation; PSB, prosocial behavior - sharing resources (*^∗^p* < 0.05, ^∗∗^*p* < 0.01, ^∗∗∗^*p* < 0.001).

The variables were as in Study 1 (Cronbach’s alphas for reliability are shown in **Figure 4**), with the addition of three new variables considered relevant to the inclusion/exclusion context.

##### Rejection sensitivity

[*M* = 3.83, *SD* = 1.45, range (1.00-7.00); *M*_included_ = 3.88, *SD* = 1.44, range (1.17-7.00); *M*_excluded_ = 3.77, *SD* = 1.46, range (1.00-6.67)]. Rejection sensitivity was measured with the six-item Hypersensitivity to Social Rejection scale (Ronen and Baldwin, 2010; e.g., ‘If someone doesn’t seem to like me I think about it for the rest of the day’), with responses given on a 7-point Likert scale.

##### Anger

Anger was assessed before and after the manipulation using a three-item (e.g., ‘angry’) short version of the anger factor of the Profile of Moods States scale (McNair et al., 1971) with responses given on a 7-point Likert scale. Descriptive statistics for anger before the manipulation were *M* = 1.52, *SD* = 0.81, range (1.00–4.33); *M*_included_ = 1.50, *SD* = 0.84, range (1.00–4.033); *M*_excluded_ = 1.53, *SD* = 0.79, range (1.00–4.00). After the manipulation the corresponding statistics were *M* = 2.05, *SD* = 1.58, range (1.00–7.00); *M*_included_ = 1.24, *SD* = 0.54, range (1.00–4.00); *M*_excluded_ = 2.90, *SD* = 1.84, range (1.00–7.00).

##### Affiliation motivation

[*M* = 5.51, *SD* = 5.51, range [1.33–7.00]; *M*_included_ = 6.16, *SD* = .74, range (3.67–7.00); *M*_excluded_ = 4.84, *SD* = 1.31, range (1.33–7.00)]. Participants’ desire to continue interacting with their group was assessed with a specially developed six-item scale (‘I wish to remain part of this group for future group tasks,’ ‘I would like to remain part of this group,’ ‘I dislike this group for future group tasks,’ ‘I would like to be fully accepted by the members of this group in the future,’ ‘I would like to be fully integrated into this group in the future,’ and ‘I would like the members of this group to accept me in the future’) to which responses were given using a 7-point Likert scale. EFA with Varimax rotation confirmed that a single factor explained 62.9% of the variance in scores.

##### Manipulation check

A manipulation check was performed after the experimental manipulation. Perceptions of inclusion and exclusion were measured with four items (‘My group members have excluded me,’ ‘My group members have included me,’ ‘I feel excluded by my group members,’ and ‘I feel included by my group members’).

#### Treatment of the Data

Sex and age were not the principal aim of our study and did not show any significant influence on the other variables of the study, and were thus omitted from all further analyses.

##### Preliminary analyses

In order to test the means and standard deviations of the variables of the study, as well as the interactions between them some descriptive analyses and correlation tests including all the variables were performed.

##### Multicollinearity tests

To detect multicollinearity we examined the correlation matrix for the independent variables, the VIF and tolerance values for all the constructs (Kline, 2005).

##### Mediation analyses

In order to confirm hypotheses 1, 2, 3, and 4 mediation analyses were computed with Amos (version 21) by following the product-of-coefficients strategy with bootstrapping to test the strength and significance of the indirect effect (Shrout and Bolger, 2002). In the present study the 95% confidence interval of the indirect effect was obtained with 2,000 bootstrap resamples.

##### Structural Equation Modelling (SEM)

In order to confirm a context-sensitive predictive model of PSB a multi-group SEM analysis (moderated analysis) was conducted with Amos (version 21) to test for the equivalence of the causal structure between the two experimental conditions; this analysis was performed according to the steps prescribed in Byrne (2009) and by using the critical ratio for differences between parameters method. To estimate the causal model the same indicators of the goodness of fit of Study 1 were used.

#### Results

##### Manipulation Check

ANOVA showed main effects of experimental condition on perception of exclusion [*F*(1,118) = 94.34, *p* < 0.001] and inclusion [*F*(1,118) = 127.31, *p* < 0.001]. Participants in the exclusion context felt more rejected [*M*_excl_ = 4.05, *SD*_excl_ = 1.88, range_excl_ (1.00–7.00); and *M*_incl_ = 1.43, *SD*_incl_ = 0.92, range_incl_ (1.00–6.00)] and less included [*M*_excl_ = 2.41, *SD*_excl_ = 1.83, range_excl_ (1.00–7.00); and *M*_incl_ = 5.75, *SD*_incl_ = 1.37, range_incl_ (2.00–7.00)] than participants in the inclusion context. We therefore concluded that the manipulation was effective.

##### Preliminary Analyses

Correlation analyses were performed to explore the relationships between all the variables in the study. As can be seen in **Tables 3** and **4**, all correlations were in the expected direction.

**Table 3:** Correlations, means, standard deviations and alpha reliabilities for all the study two variables of the general sample.

	1	2	3	4	5	6	7	8	Mean	Range	*SD*	α
(1) Prosocialness	-								6.10	(4.17–7.00)	0.62	0.87
(2) Rejection sensitivity	0.02 (ns)	-							3.83	(1.00–7.00)	1.45	0.88
(3) PS self-efficacy	0.55^∗∗∗^	-0.18^∗^	-						6.25	(4.00–7.00)	0.70	0.85
(4) Collective PS efficacy	0.03 (ns)	-0.10 (ns)	0.19^∗^	-					4.85	(1.00–7.00)	2.09	0.98
(5) Anger	-0.07 (ns)	0.29^∗∗∗^	-0.16^#^	-0.52^∗∗∗^	-				1.98	(1.00–7.00)	1.51	0.91
(6) Trust	0.20^∗^	-0.10 (ns)	0.22^∗^	0.65^∗∗∗^	-0.63^∗∗∗^	-			4.42	(1.00–7.00)	1.89	0.97
(7) SAM	0.26^∗∗^	0.06 (ns)	0.27^∗∗^	0.42^∗∗∗^	-0.36^∗∗∗^	0.60^∗∗∗^	-		5.51	(1.33–7.00)	1.25	0.87
(8) PSB	0.26^∗∗^	0.14 (ns)	0.19^∗^	0.06 (ns)	-0.08 (ns)	0.13 (ns)	0.26^∗∗^	-	2.58	(0.00–3.50)	0.78	*R* = 0.77^∗∗∗^

*#*p <* 0.09, ^∗^*p <* 0.05, *^∗∗^p <* 0.01, *^∗∗∗^ p <* 0.001*.

*For PSB’ reliability, Pearson correlation analysis was done because it is composed only by two variables. PS self-efficacy, prosocial self-efficacy; collective PS efficacy, collective prosocial efficacy; SAM, Social affiliation motivation; PSB, prosocial behavior*.

**Table 4:** Correlations, means, standard deviations and alpha reliabilities of the included and excluded samples for all the study two variables.

	Inclusion											Exclusion		
	Mean	Range	*SD*	1	2	3	4	5	6	7	8	Mean	Range	*SD*
(1) PSness	6.11	(4.17–7.00)	0.64	-	0.01 (ns)	0.49^∗∗∗^	-0.17 (ns)	0.01 (ns)	0.09 (ns)	0.25^∗^	0.28^∗^	6.08	(4.67–7.00)	0.61
(2) RS	3.88	(1.17–7.00)	1.44	0.03 (ns)	-	-0.29^∗^	-0.21^#^	0.52^∗∗∗^	-0.28^∗^	0.05 (ns)	0.07 (ns)	3.77	(1.00–6.67)	1.47
(3) PS S-Eff	6.25	(4.00–7.00)	0.72	0.61^∗∗∗^	-0.08 (ns)	-	0.10 (ns)	-0.20 (ns)	0.13 (ns)	0.23^#^	0.06 (ns)	6.24	(4.60–7.00)	0.68
(4) Coll. PS eff	6.24	(4.60–7.00)	0.70	0.54^∗∗∗^	-0.10 (ns)	0.77^∗∗∗^	-	-0.27^∗^	0.33^∗∗^	0.02 (ns)	-0.06 (ns)	3.40	(1.00–7.00)	2.08
(5) Anger	1.22	(1.00–4.00)	0.50	-0.34^∗∗^	0.07 (ns)	-0.28^∗^	-0.27^∗^	-	-0.47^∗∗∗^	-0.06 (ns)	-0.07 (ns)	2.78	(1.00–7.00)	1.78
(6) Trust	5.62	(2.67–7.00)	1.02	0.52^∗∗∗^	0.02 (ns)	0.57^∗∗∗^	0.63^∗∗∗^	-0.45^∗∗∗^	-	0.31^∗^	0.03 (ns)	3.16	(1.00–7.00)	1.76
(7) SAM	6.16	(3.67–7.00)	0.74	0.39^∗∗^	0.05 (ns)	0.51^∗∗∗^	0.45^∗∗∗^	-0.44^∗∗∗^	0.61^∗∗∗^	-	0.36^∗∗^	4.84	(1.33–7.00)	1.31
(8) PSB	2.63	(0.00–3.50)	0.79	0.24^#^	0.21 (ns)	0.29^∗^	0.22^#^	-0.03 (ns)	0.24^#^	0.14 (ns)	-	2.52	(0.00–3.50)	0.77

*^#^*p <* 0.09, ^∗^*p <* 0.05, ^∗∗^*p <* 0.01, ^∗∗∗^*p <* 0.001*.

*Upper triangle shows the excluded sample correlations (*N* = 61); lower triangle shows the included sample correlations (*N* = 58)*.

*For PSB’ reliability, Pearson correlation analysis was done because it is composed only by two variables. PSness, prosocialness; RS, rejection sensitivity; PS S-Eff, prosocial self-efficacy; coll. PS Eff, collective prosocial efficacy; SAM, Social affiliation motivation; PSB, prosocial behavior*.

##### A Context-Sensitive Predictive Model of Prosocial Behavior

To detect multicollinearity we examined the correlation matrix for the independent variables; the lack of high correlation coefficients (i.e., 0.85 or greater) indicated that collinearity was not a problem (Kline, 2005). As indicated in **Table 3**, the highest correlation coefficient was between prosocialness and prosocial self-efficacy (*r* = 0.65). Next we checked VIF and tolerance values for all the constructs. All VIF values were less than 5.0 (range: 1.070-2.415) and all tolerance values were between 0.10 and 1.0 (range: 0.414-0.934) so we can be confident that the data were not affected by multicollinearity (Kline, 2005).

Bootstrapping analyses were performed with Amos 21 to test hypotheses about mediation of relationships involving PSB (H1, H2, H3, and H4). Most hypotheses were confirmed; the exception was H4b, that affiliation motivation mediates the relationship between rejection sensitivity and PSB (**Table 5**).

**Table 5:** Type of mediation observed.

Hypotheses	Direct Beta without Mediator	Direct Beta with Mediator	Indirect Beta
H1: RS → PS self-efficacy → collective PS efficacy	-0.10 (ns)	-0.07 (ns)	-0.03ˆ*
H2a: RS → anger → trust	-0.03 (ns)	0.07 (ns)	-0.10ˆ**
H2b: PS self-efficacy → anger → trust	0.10 (ns)	0.08 (ns)	0.14ˆ*
H2c: collective PS efficacy → anger → trust	0.64ˆ***	0.42ˆ***	0.22ˆ***
H3a: PSness → trust → SAM	0.25ˆ**	0.16ˆ*	0.09ˆ**
H3b: collective PS efficacy → trust → SAM	0.49ˆ***	0.08 (ns)	0.33ˆ***
H4a: PSness → SAM → PSB	0.26ˆ**	0.20ˆ*	0.05ˆ**
H4b: RS → SAM → PSB	0.14 (ns)	0.13 (ns)	0.02 (ns)
H4c: PS self-efficacy → SAM → PSB	0.19ˆ*	0.12 (ns)	0.06ˆ**
H4d: collective PS efficacy → SAM → PSB	0.06 (ns)	-0.06 (ns)	0.12ˆ**
H4e: Trust → SAM → PSB	0.13 (ns)	-0.04 (ns)	0.17ˆ**

*Direct and indirect effects in proposed mediation relationships. PSness, prosocialness; RS, rejection sensitivity; PS self-efficacy, prosocial self-efficacy; collective PS efficacy, collective prosocial efficacy; SAM, social affiliation motivation; PSB, prosocial behavior. The first column is a statement of the mediation hypothesis. The second column presents the regression weight for the direct association between the independent variable (IV) and the dependent variable (DV) before controlling for the effects of the putative mediator (M). The third column presents an estimate of the standardized direct effect of the IV on the DV after controlling for the effects of the putative M. The fourth column presents an estimate of the standardized indirect effect of the IV on the DV after controlling for the effects of the putative M in bootstrapping analysis. ^∗^*p* < 0.05, ^∗∗^*p* < 0.01, ^∗∗∗^*p* < 0.001*.

Multi-group structural equation modeling (SEM) was performed to confirm the context-sensitive predictive model of sharing resources PSB. The model was a good fit to the data [χ^2^(15, *N* = 119) = 7.46, *p* = 0.94, *RMSEA* = 0.01, 95% *CI* [0.01,0.02]; *CFI* = 1.00, *GFI* = 0.98]. Comparison of the well-fitted baseline unconstrained model [χ^2^(30, *N* = 119) = 27.36, *p* = 0.60, *RMSEA* = 0.01, 95% *CI* [0.01,0.06]; *CFI* = 1.00, *GFI* = 0.95] with the well-fitted fully constrained model [χ^2^(43, *N* = 119) = 55.09, *p* = 0.10, *RMSEA* = 0.05, 95% *CI* [0.01,0.08]; *CFI* = 0.95, *GFI* = 0.90] using the chi-square comparison test indicated a difference between the inclusion and exclusion groups [Δχ^2^_(13)_ = 27.73; *p* > 0.01]. The critical ratio for differences between parameters method revealed groups differences in the rejection sensitivity→anger path (ß_exclusion_ = 0.59, *p* > 0.001; ß_inclusion_ = 0.01, *ns*; *z* = 4.05, *p* > 0.01) and the prosocial collective efficacy efficacy→trust path (ß_exclusion_ = 0.19, *p* > 0.05; ß_inclusion_ = 0.68, *p* > 0.001; *z* = -2.66, *p* > 0.01). **Figure 4** represents the general model for the combined sample.

#### Discussion

All the variables analyzed contribute to a predictive model of PSB — valid for both excluded and included individuals — in which prosocialness and affiliation motivation act as direct predictors of PSB. Most of the paths in the Study 1 model were confirmed. The disappearance of two of the relationships found in Study 1 — between (1) prosocial self-efficacy and PSB and (2) trust and PSB — might be due to the incorporation of affiliation motivation, which acted as a mediator of those relationships, such that there were no longer direct associations between the independent variables and PSB. The model was valid for both included and excluded individuals although there were two path differences. First, rejection sensitivity only predicted anger in the context of exclusion; this is consistent with previous reports that rejection-sensitive individuals only react with anger when they feel rejected (Downey et al., 2000; Levy et al., 2001; Luterek et al., 2004). Second, there was a stronger association between collective prosocial efficacy and trust in the context of inclusion. Individuals who were confident in the ability of their group to act prosocially trusted their partners more, particularly when they felt included in the group. This provides some evidence, albeit weak, that exclusion reduces trust (Twenge et al., 2007).

In line with previous studies (Eisenberg et al., 2002; Carlo et al., 2003) we found that prosocialness was a direct determinant of PSB and that individuals tend to behave in accordance with their dispositions (Heider, 1958). Affiliation motivation was also a direct predictor of PSB. The desire to maintain social contact motivates individuals to behave in a prosocial and friendly way in order to achieve affiliation (Baumeister and Leary, 1995; Zaskodna et al., 2013).

There was evidence for all the hypothesized mediation relationships except for the mediation of the relationship between rejection sensitivity and PSB by affiliation motivation. Nevertheless, the predictive model confirms that, as expected, rejection sensitivity negatively predicted affiliation motivation (Shipley and Veroff, 1958; Maner et al., 2010), which in turn was a positive predictor of PSB (Baumeister and Leary, 1995; DeWall and Richman, 2011; Zaskodna et al., 2013).

Prosocial self-efficacy mediated the association between rejection sensitivity and collective prosocial efficacy. The more sensitive individuals are to social rejection, the more likely they are to feel rather incapable of PSB (Butler et al., 2007), and also to feel that their group is relatively incapable of PSB (Fernández-Ballesteros et al., 2002).

Anger runs as a mediator between three different links. It mediated the relationship between rejection sensitivity and trust; rejection sensitive individuals tend to report greater anger (Downey et al., 2000; Levy et al., 2001) and in turn to have less trust in others (Jones and George, 1998). Anger also mediated the relationships between self- and collective prosocial efficacy and trust. In other words when individuals feel that they or their group are relatively incapable of PSB they tend to report greater anger (Bandura, 2000; Valentino et al., 2009) and to trust their interaction partners less (Jones and George, 1998).

Trust mediated two relationships. It was a partial mediator of the prosocialness-affiliation motivation association. The more individuals have a prosocial tendency, the more they trust others (Nooteboom and Six, 2003), and in turn the more they wish to affiliate with their group (Patrick et al., 1997), probably because prosocial individuals tend to feel that others resemble them, expect some reciprocity, and consequently trust them (Levenson and Ruef, 1992) and wish to keep in contact with them. Trust implies an expectation that others will operate with goodwill and therefore motivates individuals to engage with others thus producing a social orientation; trust also promotes closeness which is the starting point for friendships (Welch et al., 2005). It therefore seems logical that trust would increase affiliation motivation (Green and Brock, 1998). Second, we found that trust fully mediated the prosocial collective efficacy-affiliation motivation association; individuals who felt their group was capable of PSB were more likely to trust group members (Sapouna, 2010) and in turn more motivated to affiliate with them (Patrick et al., 1997).

Affiliation motivation mediated four different relationships. It was a partial mediator of the prosocialness-PSB relationship. As a personal trait that includes the tendency to take care of others prosocialness (Caprara et al., 2000) obviously increases (1) the desire for positive interaction with the others, i.e., affiliation motivation (Hill, 1987) and (2) PSB; this is consistent with Eisenberg et al. (2002), Carlo et al. (2003) and with the theory that individuals tend to behave in a way which is consistent with their thoughts, beliefs and attitudes (Heider, 1958).

Affiliation motivation also mediated the associations between prosocial efficacy—both self and collective—and PSB. Our results showed that individuals who felt that they and their group were highly capable of PSB were more motivated to affiliate with others (Patrick et al., 1997), probably because perceiving oneself or one’s group as prosocial motivates individuals to develop positive interpersonal relationships and maintain social contacts. A higher desire to maintain social contact in turn results in more PSB, probably because, as Baumeister and Leary (1995) and Zaskodna et al. (2013) argued, individuals with high affiliation motivation behave in a friendly way in order to maintain social contact and avoid exclusion.

Affiliation motivation also mediated the relationship between trust and PSB. Trusting individuals were more likely to desire social contact (Patrick et al., 1997) and in turn more likely to engage in PSB (Baumeister and Leary, 1995; Zaskodna et al., 2013).

Note that the mediation analyses indicated that two variables, prosocialness and affiliation motivation, were direct predictors of PSB. In addition path analysis confirmed the direct and indirect predictive relationships detected in the mediation analyses.

## General Discussion and Concluding Remarks

The validity of our model in the contexts of inclusion and exclusion indicates that psychosocial interventions designed to foster prosocialness, individual and collective prosocial efficacy, trust and affiliation motivation, as well as interventions to decrease negative affect, have the potential to promote PSB in both excluded and included individuals. The differences in relationships in the two contexts suggest, moreover, that psychosocial interventions could be used to (1) mitigate the negative impact of rejection sensitivity, especially in individuals who feel ostracized and (2) increase trust, especially in excluded individuals.

Affiliation motivation is possibly the most interesting of the mediators we identified. In Study 1 we demonstrated that trust tends to engender PSB, whilst in Study 2 we demonstrated that this relationship was mediated by affiliation motivation. It is possible that trust enhances the probability that someone will act prosocially (Rotenberg et al., 2005) precisely because it enhances intrinsic motivation to affiliate (Parks et al., 1996; Green and Brock, 1998). This might explain why Twenge et al. (2007) failed to show that trust mediated the effect on PSB — because affiliation motivation mediates the trust-PSB relationship. We also found that affiliation motivation mediated the relationship between PSB and most of the predictor variables we investigated. This pattern of results suggests that affiliation motivation may be a predictor of PSB in both included and excluded individuals and it follows that practitioners should take special care to enhance individuals’ affiliation motivation as a means of fostering PSB. In this context we suggest that it would be useful to promote broad, strong social networks.

### Limitations and Future Directions

Although this study has implications for our understanding of the psychosocial determinants of PSB it is important to highlight its limitations. The data for both studies were from a student sample with a majority of women so care must be exercised in interpreting the findings and they may not generalize to the wider population. There is no reason to believe, however, that relationships investigated in these studies would be different in the student and general populations. It would nevertheless be interesting to replicate this study in a larger sample that was representative of the general population; such a study would allow the investigation of potential sex and age effects.

In these studies the possible interactions were limited; participants were members of a group of (fictitious) strangers and all interactions took place online. We also cannot be sure that the Cyberball task represents a good proxy for real world inclusion and exclusion contexts. For these reasons our results may not generalize to genuine personal relationships and real world social exclusion. In this context it is relevant that humans tend to act for the benefit of close relations (Olson and Spelke, 2008; IJzerman et al., 2015). Iannone et al. (2014) showed that being excluded by two people who were stranger to each other made participants feel worse than being excluded by two people who were friends with each other. We also note that whilst laboratory studies have shown that exclusion at the hands of an out-group is painful (Williams et al., 2000; Smith and Williams, 2004), even if the out-group is despised (Gonsalkorale and Williams, 2007), a study of real life exclusion showed that rejection by people to whom one feels close is more painful that rejection by strangers or acquaintances (Nezlek et al., 2012). Future research should investigate how the relationships we have identified are influenced by the ecological validity of the exclusion manipulation and the strength of the social relationship between an individual and the group which excludes him or her.

Similarly, we can wonder about the external validity of the PSB measure, and whether the prisoner’s dilemma game is useful in thinking about real world situations. Note that different studies have corroborated the external validity of the public good games (Franzen and Pointner, 2013; Stoop, 2014; Goeschl et al., 2015; Rommel et al., 2015). In a recent study, Franzen and Pointner (2013) have demonstrated that in lab behavior is related to PSB in the field—these authors used a measure of PSB with a dilemma game similar to the one we have used in this experiment. Moreover, recently Goeschl et al. (2015) have shown that the prisoner’s dilemma game is related to PSB (giving money to reduce CO^2^ emissions) in the field. Thus, research is showing some evidences of external validity of the prisoner’s dilemma game, and there is no reason to believe that the measure used in this experiment to assess PSB (the prisoner’s dilemma game) does not have ecological validity. Nevertheless, it would be interesting in future research to analyze the applicability of the game to the real world.

Another potential limitation is that our outcome variable was related to the winning or sharing of a monetary reward whilst PSB encompasses a wider spectrum of interpersonal interactions and behaviors (Weinstein and Ryan, 2010). In future research it would be interesting to measure a broader range of PSB, including helping behavior, altruism, cooperation, and solidarity as well as the sharing of resources (Weinstein and Ryan, 2010).

## Conclusion

As all the variables we investigated were related to PSB practical interventions to increase PSB should be designed to (1) promote a more prosocial disposition, encourage individuals to perceive themselves as capable of PSB, encourage trust in others and increase affiliation motivation and (2) work on negative feelings and on the tendency to dread rejection to reduce their negative impact on PSB. Romero-Canyas et al. (2010a), suggested that the vicious cycle involving rejection sensitivity and exclusion could be interrupted by promoting general self-regulatory skills and experiencing supportive relationships; we suggest that a similar strategy could be used to promote the motivational determinant of PSB.

## Conflict of Interest Statement

The authors declare that the research was conducted in the absence of any commercial or financial relationships that could be construed as a potential conflict of interest.
